# Multi-parametric sensing by multi-channel molecular fluorescent probes based on excited state intramolecular proton transfer and charge transfer processes

**DOI:** 10.1016/j.bbadva.2023.100094

**Published:** 2023-06-01

**Authors:** Vasyl G. Pivovarenko

**Affiliations:** Department of Chemistry, Kyiv National Taras Shevchenko University, 01033, Kyiv, Ukraine

**Keywords:** Fluorescent multi-parametric multi-channel probes, Peptides, Lipid membranes, Nucleic acids, Atp, Cation recognition, Local concentration measurements, Ratiometric response

## Abstract

Considering the applications of fluorescent probes and the information they provide, their brightness of fluorescence and photostability are of paramount importance. However, in the case of steady-state fluorescence spectroscopy and fluorescence microscopy, the amount of information can be increased by the application of multi-channel probes, via a multi-band fluorophore introduced in the probe molecule. In most cases, the use of such a multi-band (or multi-channel) fluorophore can also be combined with the concomitant introduction of one or several analyte receptors. Most often, the design of ratiometric probes with multi-band fluorescence emission are based on phenomena such as photoinduced intramolecular charge transfer (ICT) or excited state intramolecular proton transfer (ESIPT). Although ICT probes were up to recently the most popular, ESIPT probes and among them 3-hydroxyflavone derivatives, were shown to be the most productive. Several general problems were resolved by this family of probes, as for example the measurement of local dielectric constant, local H-bond accepting ability, water local concentration and ATP concentration in small volumes.

Incorporation of such multi-channel probes into lipid membranes allowed to measure the different membrane potentials and to detect cell apoptosis. Also, it enabled to recognize and characterize the rafts formation in different lipid bilayers and peculiar features of the charged membrane interface. Such probes are also able to provide a concentration-dependent fluorescence signals upon binding of *H*^+^, Mg^2+^and Ba^2+^ions, and thus to recognize these different cations.

The multi-channel probes are effective tools in the study of interactions of macromolecules such as peptides, proteins and nucleic acids. The most useful feature is that they inform simultaneously about several physical parameters, in this way giving a better insight in the investigated system. Thus, by comparing the reviewed probes with other modern fluorescent approaches, it can be concluded they are more informative and accurate tools.

## Highlights

• Information transmission channels in molecular fluorescent probes of different composition.
• Intramolecular charge transfer and excited-state intramolecular proton transfer effects as methods of increasing the number of probe information channels.
• Highly selective detection of ATP and peptide-lipid, peptide-protein, peptide-DNA, DNA-DNA, DNA-RNA interactions.
• Examples of fluorescent recognition of cations of different radii and a wide-range pH indicator.
• Separate measurement of local polarity and local concentration of H-bond donors in the environment.
• Mesurement of local water concentration in bio-objects.

## Graphical abstract

**Figure:**
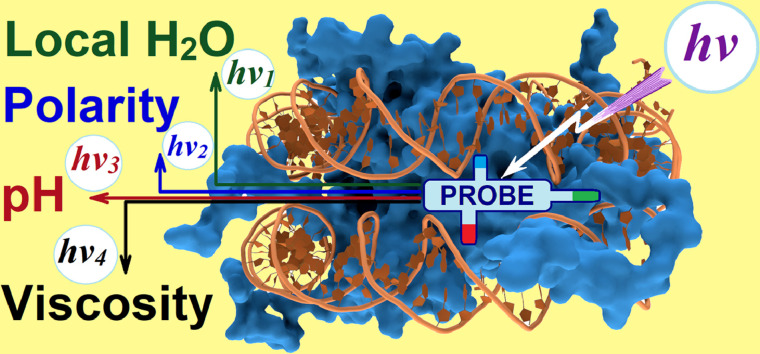

## Introduction

At home and at work - everywhere we are now surrounded by multi-channel information transmitting devices, e.g. televisions, laptops, smartphones, etc., with numerous systems for organizing information flows. What are the prospects and trends on the organization of the smallest multi-channel devices - the fluorescent probes of a single molecule sizes - this problem will be discussed below.

Fluorescence techniques are now widespread and effective tools for chemical and biological applications, especially in the study of objects at the molecular level [1], [2], [3]. Due to their sensitivity, fluorescence methods occupy a first-range position for the investigation of complex molecular systems. Taking benefit from the technical progress, scientists have now the possibility to register single light quanta from volumes lesser than 0.5 femtoliter, in the femtosecond timescale. In such cases where the information should be obtained from very small volumes, fluorescent probes were shown to be the most effective tools. These individual molecules or their complexes act as transmitters of information, where its elements are the wavelength of absorbed or emitting radiation, its intensity or intensity ratio, anisotropy or fluorescence lifetime.

Of course, intensiometric probes with the brightest fluorescence are the most popular now. However, on several reasons the structure of their fluorophore limits the possibilities of molecular design. The main problem is they are intensiometric, i.e. a single-channel and the least informative, reporting only the single studied parameter of the object. Nowadays, the increased sensitivity of devices for counting photons provides new opportunities for the use of fluorescent probes with increased informativeness, despite their somewhat lower brightness. This is especially important for the experiments in microheterogeneous solutions, e.g., in cells and tissues.

Why is the informativeness of the probe so important and how best to organize the flow of information in steady-state spectroscopy? Every scientist will agree that the adequacy of a signal to event and its unambiguity, i.e., the fight against artifacts, is in the first place in experiment. The most vulnerable to generation of artifacts are intensiometric probes, in which a useful signal is transmitted through a single information channel - by changing the intensity of fluorescence. A change in intensity means a turning off/on a separate mechanism of fluorescence quenching during the probe - analyte interaction. Unfortunately, there are several quenching mechanisms known, and all of them may drive the probe fluorescence in solutions independently on probe - analyte interaction event. As a result, an experiment may imperceptibly enter the zone of obtaining artifacts. E.g., in microheterogeneous biosystems, a false signal is often may be caused by a changes in local viscosity of the probe surroundings or by changes in local concentration of water due to an unpredictable event.

In order to prevent artifacts, it is important to simultaneously obtain data on several parameters of the molecular environment, such as composition of probe surrounding, including the presence of analyte, local viscosity, polarity, etc. In a steady-state fluorimetry it can be performed by creating of several information channels, such as signal intensity at several wavelengths. Here there is a requirement to divide the data on physical events by information channels. So that the influence of each physical parameter would be different part of content in each of the organized information channels. Currently, such direction is intensively developing in the aspect of two-channel, or ratiometric probes [4], [5], [6]. This is because most often in experiments scientist seeks to record the change of only single physical parameter at the probe location or the concentration of only single analyte in the object. The investigated parameter is calculated from the ratio of fluorescence intensities at two wavelengths *I_λ1_*/*I_λ2_*, and its changes is based on the change of this ratio. That is what gave rise to the name of ratiometric method. Sometimes intensity ratio *I_λ1_*/*I_λ2_* may be transformed into band maximum position parameter, but they are not the same in complex cases. The creation and widespread application of ratiometric probes, where the fluorescence intensities at two wavelengths always presents an analytical signal with internal calibration can be considered as a first step on the multi-channel probes evolution.

Ratiometric probes became popular after their fluorescent cores were synthesized, which was the first solvatofluorochromic dyes that change the color of fluorescence in the media of different polarity. In fact, there the change in color has always been an influence of several environmental parameters, such as intermolecular electric fields, the ability of atoms to donate and accept H-bonds, and viscosity of medium in part. The most popular representatives of this class, which have been widely used for a long time, include 5-(dimethylamino)naphthalene-1-sulfonate (DNS), tryptophan and PRODAN derivatives. They are used on practice until now, despite that recently a number of dyes with more pronounced solvatofluorochromism, bright red fluorescence and greater photostability appeared [6], [7], [8], [9].

All solvatofluorochromic dyes have a specific structure. Their molecules present the dipoles of high polarizability, composed of electron donating and electron withdrawing substituents on their poles. Their polarization increases in the excited state due to the increased mobility of electrons. Thus, the bound ion or surrounding molecules have the possibility to further increase the dye polarization during the relaxation time in S_1_ state and to shift the emission band down towards higher wavelengths, in special cases with small changes of fluorescence brightness. This corresponds to the Intramolecular Charge Transfer (ICT) effect [4,5]. A more polar solvent gives rise to a larger red shift. In the case of probe-ion binding, the direction of the shift depends on the location of receptor unit in the probe molecule and on the charge of the ion (see examples below).

Dipolar composition of solvatofluorochromic dyes enables ion sensing once an analyte receptor unit has been added close to an end of the dipole. In this case, free and ion-bound species of the probe, both fluorescent, are giving two separate bands in the spectrum due to the analyte influence on the energy gap value between S_1_ and S_0_ states.

During the last decades many molecules have been elaborated based on ICT principle [10], [11], [12]. All of them give a ratiometric response in the presence of cationic analytes for a wide range of concentrations, depending on the cation receptor unit design. The most popular ones have found application in cell biology, e.g. for the measurement of physiological levels of pH, Ca^2+^, Zn^2+^, Mg^2+^, Na^+^ and *K*^+^ concentrations.

Until now, considerable difficulties concerning synthesis of the receptors for anions remain pending. They have been successfully resolved for the sensing of several anions [13], including multi-charged mononucleotides [14].

Thus, elaborated probes based on ICT principle have convincingly shown their advantages over intensiometric ones in cases where the analyte is a polar molecule or ion. At the same time, their individual shortcomings were revealed. For example, when detecting the local polarity of the local surroundings by them, it is possible to obtain only the parameter of so-called "empirical polarity", which is actually the total effect of electric fields and H-bond formation on fluorescence of the probe. This drawback is the result of low resolution of the fluorescence bands originating from free probe molecules and its complexes with several close by properties analytes, which indicates a limitations in the applications of the ICT principle. For the organization of three or more channels of information transmission, it is worth applying other principles based on the properties of excited molecules.

New fluorophores for ratiometric sensing capable of operating in a wide range of parameters of surroundings were elaborated. On this course, a new type of probes based on the Excited State Intramolecular Proton Transfer (ESIPT) principle has demonstrated its advantages in several aspects and many cases of various analytes [1,12].

What are the advantages of the probes those work on the mechanism of the ESIPT reaction? First is an increased number of information transmission channels. Depending on the experimental conditions, three bands can be formed in the fluorescence spectrum of such probe, which are usually well separated. There are the excited parent molecule, its tautomer and its deprotonated (anionic) or protonated (cationic) forms. By installing electron-donating and electron-accepting substituents on different ends of the molecule, it is possible to increase its polarization, which will ensure the functioning of the probe by the ICT principle as well. Simplicity in the synthetic modification of the probe molecule can provide a number of additional advantages in applications. The positions of the bands mentioned above, as well as the pairwise ratios of their intensities, make it possible to organize six full-fledged channels of information with internal calibration of a signal in fluorescence emission spectra. It is possible in addition to organize as minimum one channel with internal calibration in fluorescence excitation spectra.

This review scope is to consider the possibilities of ICT and ESIPT probes application in the directions of study of different interactions in solutions on the scale of molecules and their aggregates. The probe design for ratiometric detection of local physical parameters of solutions, such as dielectric polarity, pH level, water content, or that of other analytes of more complex structure is discussed. For this purpose, the probes based on 3-hydroxyflavones and 3-hydroxyquinolones, which are among the most convenient and advanced ones in terms of fluorescence brightness, amplitude of response and molecular structure tuning capabilities are presented.

## Section 1. multi-channel probes based on ict effect and their limitations

### Functional elements of fluorescent probes. design principles of multi-channel probes

**Fig. 1:**
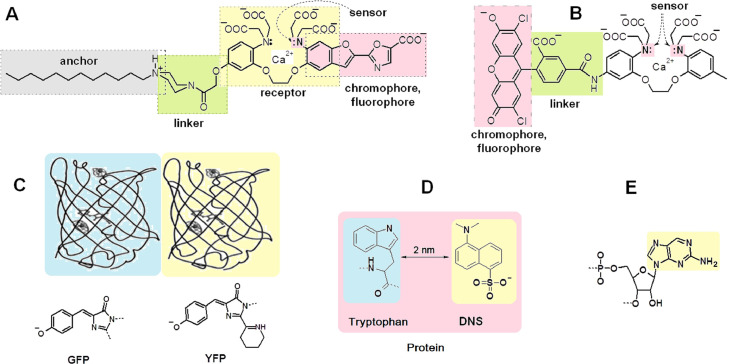
Functional elements of fluorescent probes. A) Example of a membrane interface calcium indicator [15]. B) Indicator Fluo-3 used to determine Ca^2+^ concentration in cells [16]. C) Schematic view of green (GFP, on blue, energy donor unit) and yellow (YFP, on yellow, energy acceptor unit) fluorescent proteins and structure of their chromophore-fluorophore units [17]. D) Another example of remote units of chromophore and fluorophore [18]. E) Fluorescent 2-aminopurine, with required spatial structure to mimic and replace adenine nucleic base [19].

Despite fluorescent probes are small molecules, they should be considered as complex devices consisting of several functional elements (Fig. 1A,B). Each probe contains a chromophore, the light absorbing unit, and a fluorophore, the light emitting unit. They both usually are the ground and excited states of the same moiety, but sometimes they can be located in different parts or even in separate molecules, e.g. in fluorescence resonance energy transfer (FRET) pairs (Fig. 1C,D). In addition, the probe frequently contains chemical groups (e.g., carboxylates, sulfonates, peptide chains etc.) which prevent aggregation phenomena and fluorescence quenching by water. The probe should also contain a receptor unit responsible for the analyte binding and binding selectivity versus other competing objects. A sensor unit switches the color of fluorescence at presence of analyte. The probes qualified as fluorescent labels should contain two additional elements, an anchor and a linker. The purpose of the anchor is to immobilize the probe covalently or noncovalently on the studied system. The linker (spacer) regulates the distance and probe orientation relatively to the system in an adequate way for a proper function of the probe. A strict spatial adequacy is required concerning the size, shape and composition for probes mimicking nucleotides. Thus, it is important in the design of new probes for all these requirements to be counted. According to such a simplified view, a multi-channel probe for the detection of several parameters of surrounding should be equipped with receptors and sensors for several analytes at different locations of the fluorophore, so that binding of each analyte generates a different analytical response.

For example, in a membrane interface calcium indicator (Fig. 1A) the anchor provides a noncovalent binding to the membrane. The linker ensures a ∼0.5 nm distance of the receptor from the membrane surface. The receptor is designed for selective binding of Ca^2+^ (working range: 2·10^-8^–2·10^−6^ M Ca^2+^ at pH 6–8). The chromophore-fluorophore unit shows solvatochromism resulting in a shift of both absorption and emission bands when Ca^2+^ is bound to the receptor. The role of sensor is played by the nitrogen atom, whose lone electron pair can be withdrawn by the chromophore or by Ca^2+^ bound to the receptor, thus inducing a shift both absorption and emission spectra. All carboxylate anions provide protection of the receptor against the aggregation with lipids or proteins. In the indicator Fluo-3 used to determine Ca^2+^ concentration in cells (Fig. 1B) the both nitrogen atoms in the receptor unit are sensors of cation. Being free of cation, their lone electron pairs switch off the fluorescence in a result of photoinduced electron transfer to the fluorophore unit. Such probe shows a single band of calcium complex in the fluorescence spectrum and therefore belongs to single-channel reporters. In the green (GFP) and yellow (YFP) fluorescent proteins pare (Fig. 1С) peptide chains around provide the chromophore rigidity and protection against quenching and photo-oxidation, thus allowing stable and high fluorescence quantum yields. At short distances (≥2 nm), GFP can act as a donor and YFP as an acceptor in excitation energy transfer process. At high distances GFP and YFP function as independent probes with green and yellow fluorescence. Another example of remote units of chromophore and fluorophore is a tryptophan containing protein (e.g. albumin) binding the DNS probe (Fig. 1D). As a result the intrinsic UV fluorescence of tryptophan transforms into green fluorescence of DNS because of excitation energy transfer from the tryptophan to DNS. Fluorescent 2-aminopurine (Fig. 1E) possesses required spatial structure to mimic and replace adenine nucleic base [19].

**Fig. 2:**
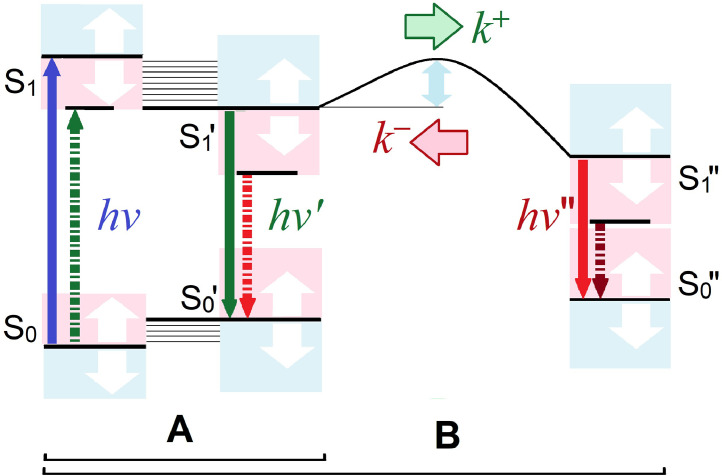
(A) Generation of new bands in the fluorescence excitation and emission spectra after formation of probe-analyte complex, presented by the new distances between the S_0_ and S_1_ of the chromophore and S_0_'-S_1_′ levels of the fluorophore. (B) Additional possibilities for new band generation in the case of chemical reaction or excitation energy transfer processes.

The creation of new information channel that reproduces the probe – analyte binding in steady-state fluorimetry consists in the generation of new band belonging to the probe-analyte complex in the fluorescence excitation or emission spectrum. Regarding the multi-channel sensing each analyte should give separate band in the spectrum. Using the Jablonsky diagram it is most convenient to consider the principles of operation of a multi-channel probe (Fig. 2). Since the binding of a polar or charged analyte to the receptor changes the energy of the ground and excited states, this will cause a change in the distance between the S_0_ and S_1_ levels of the chromophore upon absorbing a photon and the fluorophore when emitting it. Depending on the charge of the analyte and the location of the analyte receptor in the probe molecule, binding to the analyte will cause the appearance of a new band in the fluorescence spectrum, shifted to the long or short wavelengths. Localization of a negative charge near the electron-donating end of the chromophore (- close to +) or positive one near the electron-accepting end of the chromophore (+close to -) will generate a new band of the probe-analyte complex in the long-wavelength region of the fluorescence excitation and emission spectra (Fig. 2A). Other combinations (+ close to + or - close to -) will generate a new band of probe-analyte complex in the short-wavelength region of the spectrum, because the S_0_-S_1_ and S_0_'-S_1_′ energy distances will increase.

On this course the solvatofluorochromic dyes being the molecular dipoles of high polarizability may present a suitable core in multi-channel probe creation, because the distance between bands of free probe and probe-analyte complex will be higher in this case due to ICT effect [10], [11], [12]. A couple of such examples concerning the detection of one and more cations or one and more H-bond donor molecules will be discussed in the Chapter 2. The next improvement was done by creation of the probes based on the principles of photochemical reaction or excitation energy transfer processes (Fig. 2B). In these cases new excited-state specie appears in the solution, which possesses different fluorescent properties both in free state and in a complex with analyte. This opens a possibility to collect information by several new channels presented by intensity, position and halfwidth of the species’ fluorescence band. The mathematical description of the formation of the complex can be more complicated, since both the photochemical reaction [20,21] and the excitation energy transfer [22] are characterized by the rate constants of forward (*k^+^*) and reverse (*k ^−^*) reactions, and also by the rate constants of radiative and non-radiative transitions to the corresponding S_0_ states. In each case the mechanism of transition are in dependence on chemical structure of fluorophore, temperature and nature of surrounding molecules [21], [22], [23], and also on the chromophore – fluorophore distance for the probes, working by excitation energy transfer principle [24]. Examples of applications of the probes working on the Excited State Intramolecular Proton Transfer (ESIPT) reaction principle can be found in reviews [[2], [3], [4],[11], [12], [13], [14]] and books [1a,b]. In Chapters 3–16 of present review appropriate examples of the ESIPT probes will be discussed for demonstration of their application to detection of analytes of different nature. Initially, the determination of general parameters of the environment, such as local dielectric polarity, local concentration of hydrogen bond donors or acceptors (in particular – water), the detection of various cations and their nature or concentration will be discussed in parts 3–8, Sections 2 and 3. Then, the most difficult cases of analyte detection, such as tetra-charged ATP anion, and anion effects in lipid membranes as well as detection of peptide-DNA, peptide-RNA, peptide-lipid membrane, DNA-DNA, DNA-RNA interactions, etc. by the fluorescent probes will be considered in Section 4. We demonstrate that the probes based on 3-hydroxyflavone and 3-hydroxyquinolone are the best for the control over several analytes or over individual parameters of local probe surrounding to be divided between separate information channels, which are presented by the individual fluorescence bands of the probe or by the changes of their positions in the spectrum.

### Intramolecular charge transfer probes

**Fig. 3:**
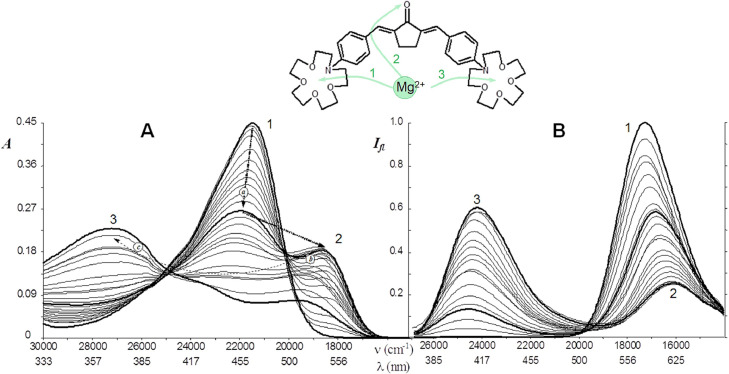
Absorption (A) and emission spectra (B) of bis-crown ketocyanine dye in acetonitrile with increasing concentration of Mg^2+^ cations [25]. The figures show the spectra of the free probe (1) and the complexes of composition 1:2 (2) and 1:3 (3).

To the best of our knowledge, one of the first examples of multi-channel probe with several receptors of cations is a bis-crown ketocyanine dye 1 (Fig. 3). This dye has a carbonyl group and two macrocyclic cation chelators with appropriate affinity to Na^+^, Ba^2+^, Ca^2+^, Mg^2+^ and La^3+^cations in aprotic solvents [25], [26], [27], [28]. It contains a partially conjugated bichromophore system with two positive poles (aryl amine moieties) and one negative pole (carbonyl group). In S_1_ state, due to intramolecular charge transfer, the bichromophore system transforms into an one with stronger conjugation, that results in a considerable Stokes' shift of fluorescence band due to decrease of energy of S_1_ state. Binding of cation to the carbonyl oxygen atom further increases the conjugation giving the additional red shift of the band. The binding of cation to first macrocyclic chelator gives raise the energy of S_1_ state, leading to a blue shift of the band. In addition, partial cation ejection from macrocycle is observed by the decrease of stability constant of the complex in S_1_ state.

As a result, such a specific probe structure gives huge shift amplitudes both in absorption and emission spectra upon binding different cations. This allows recognizing the complete sequence of binding steps for three Mg^2+^ cations and their binding positions. Moreover, it allows to distinguish between the binding of Mg^2+^ and Ba^2+^ cations by the shape of spectrum, as well as protonation by total fluorescence quenching [25,27]. For example, an increase in the concentration of Mg^2+^ cations in solution causes a consistent decrease in intensity of the absorption band and the appearance of a long-wavelength band (2), indicating the coordination of the first cation in the macrocycle, and the second one - with the lone electron pair of the carbonyl group (Fig. 3A). In the fluorescence spectra, the binding of Mg^2+^ manifests at significantly higher concentrations of the cations (Fig. 2B). The last demonstrate that both of mentioned cation receptors in S_1_ state of the probe have a close affinity to Mg^2+^, resulting in a simultaneous drop in fluorescence intensity and band shift (2) in the spectra. At maximal concentrations of Mg^2+^, the bands of a 1:3 complex with three cations are recorded in both the absorption and fluorescence spectra (3).

In the case of Ba^2+^ cations, changes in the absorption and fluorescence spectra have another view due to a different sequence of binding of cations to receptor [25]. Thus, due to the installation of cation receptors at the opposite poles of the chromophore, the multi-parametric sensing of several cations was carried out with high amplitude of spectral effects. In all cases binding with a certain cation is reproduced as a intensity change at two wavelengths - the excitation/emission maximum of free probe and its complex with the analyte. Other examples of multiparametric probe response upon binding to various analytes are collected in the review [29].

### Detection of H-bond complexes of different stoichiometry with the ict probe

**Fig. 4:**
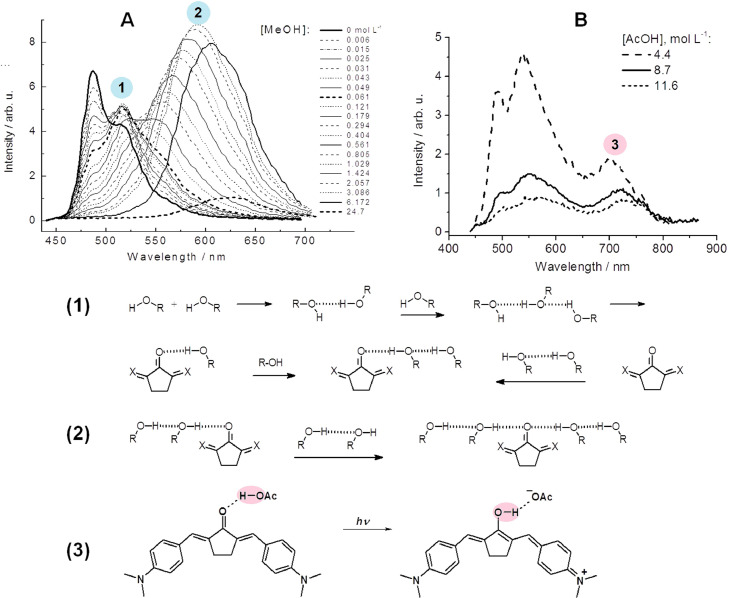
Fluorescence spectra of bis-crown ketocyanine dye in toluene: A) with increasing concentration of methanol [30]; B) with increasing concentration of acetic acid. (1) and (2): Schemes of the formation of H-bonded complexes with alcohols at stoichiometry 1:1 and 1:2. (3) Scheme of transformation of H-bonded complex with acetic acid into ion pair in S_1_ state. Complexes numbering as on panels A and B.

It was important to investigate the fluorescent response of a dye with high amplitude of intramolecular charge transfer to the hydrogen bonding with protic component in surrounding, e.g. with alcohols, water and acetic acid. The same series of ketocyanine dyes was applied in this respect [30]. Lone pairs of carbonyl oxygen of the dye serve as receptors for protic molecules. Two types of complexes with alcohols through one and two lone pairs of the carbonyl group were identified due to high amplitude of fluorescence response expressed as an additional bands in the spectrum (Fig. 4; A and (1), (2)). Moreover, fine spectral features in the regions of isoemissive points allowed to distinguish the binding with monomers and aggregates of alcohol molecules. In addition, the published earlier excited state conversion of H-bonded complex of acetic acid with ketocyanine into the ion pair also was registered [31] (Fig. 4; B and (3)).

Thus, the installation of additional analyte receptors to the solvatofluorochromic dye gives the possibility of registration of several binding events by the fluorescence spectra of the probe. The different affinity of the receptors to analyte and high response amplitude of the fluorophore provide an adequate signal about the binding event. In all cases, binding a certain cation is reproduced as a change in intensities at two wavelengths in the excitation or emission spectra of free probe and its complex or two consecutive complexes with the analyte.

Analyzing the results obtained with the help of ICT probes, one can also pay attention to their shortcomings. The main is too large band widths in the fluorescence spectra, that is associated with the heterogeneity solvate shells of free probes and probe-analyte complexes. Their high polarizability here is manifested as a disadvantage, which may complicates the application. The second negative consequence of such property is the switching of the charge transfer effect to the photoinduced electron transfer effect. The latter, as is known, is a matter of fluorescence quenching. So that many of the ICT probes of the highest sensitivity (for example, the well-known fluoroprobe) in highly polar H-bond donor media (water, methanol) or when bound to an ion specie have very low fluorescence quantum yields. Such shortcomings prompted scientists to search for other principles of operation with multi-channel probes.

## Section 2. multi-channel sensing with the esipt probes. determination of local parameters of environment

Despite their advantages, solvatofluorochromic dyes display some drawbacks as the probe fluorophores. In a result of inhomogeneous solvation of the probe-analyte specie by surrounding molecules and high sensitivity of the dye to such effect, they exhibit an increased halfwidth of the emission band. That complicates the quantitative analysis of the experimental results. As an example, in lipid membranes the fluorescense of ketocyanine probes covers the whole visible light spectrum range [32]. In this case, the same type of response of the probe to several types of intermolecular interactions in the solution becomes an obstacle in their identification. In the case of the probes applying ESIPT reaction this problem was overcome.

**Fig. 5:**
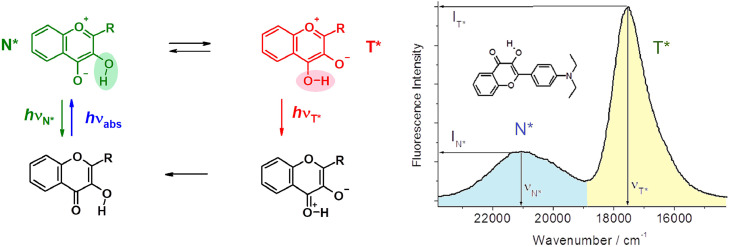
Excited State Intramolecular Proton Transfer (ESIPT) reaction in 3-hydroxyflavone (3HF) and an example of fluorescence spectrum of 3HF derivative in ethylacetate .

ESIPT reaction proceeds at close location of a proton donor and a proton acceptor groups, when the acidity of the first and the basicity of the last, both increase in S_1_ state (Fig. 5). The distance between these two groups, the spatial structure of the dye as well as the nature of surrounding molecules influence the kinetics of ESIPT reaction by the change of energy levels difference between tautomeric N* and T* states and activation energy of the reaction (Fig. 2). An increase of fluorescence quantum yield and molar extinction frequently become important for such kind of probes in solving practical problems [11,33].

On mentioned reasons, first question which occurs when designing ESIPT devices is a choice of the optimal proton transferring fluorophore. By analyzing numerous data concerning structure and properties of ESIPT dyes, we gone to the conclusion that the intramolecular H-bonding occurring in five-membered cycle of both 3-hydroxyflavones (3HFs) and 3-hydroxyquinolones (3HQs) presents an optimal configuration in respect of high fluorescence quantum yield, molar extinction and wide possibilities of tuning sensing properties. Here, the smooth tuning of fluorescence of the dye under the influence of surrounding molecules is ensured by the conformational limitations of the ESIPT system. To adjust the sensory properties of the chromophore by changing its chemical structure, a number of simple synthesis methods have been developed. Indeed, this choice appeared very productive, as evidenced by the examples of many molecules of 3HFs and 3HQs series, which will be presented and discussed thereafter.

M. Kasha and coworkers [34], [35], [36], [37] were the first to recognize the high potential of 3-hydroxyflavones in the studies of complex systems, including biological ones. With these particularly interesting molecules, they were detecting traces of water in hexane by the increased N*/T* band intensity ratio and by the appearance of a third fluorescence band [34]. Their results on the intermolecular H-bonding in rigid solvent matrix [35], on binding of 3HFs to proteins with information about the nature of the binding site environment [36,37] were also fruitful. The information was obtained through an analysis of all excitation and emission bands position and intensity. Their works can be considered as the first applications of multi-channel fluorescent probes to the study of microheterogeneous or multi-component solutions on molecular level.

### Sensing physical polarity of H-bond donor and aprotic media

In principle, 3HFs can present three bands in their emission spectra (N*, T* and anionic A* forms) and two bands in their excitation spectra, belonging to the ground states of both N and anionic forms. By combination of all these bands parameters available from steady-state spectra up to ten different information channels can be organized to analyze the data concerning the environment of the probe. This feature represents a clear advantage as compared to other probes that could be applied in different practical tasks. In this case, to simplify the processing the information obtained in the experiment, the first task of the researcher is correct selection or modification of the probe molecule structure for its special function.

**Fig. 6:**
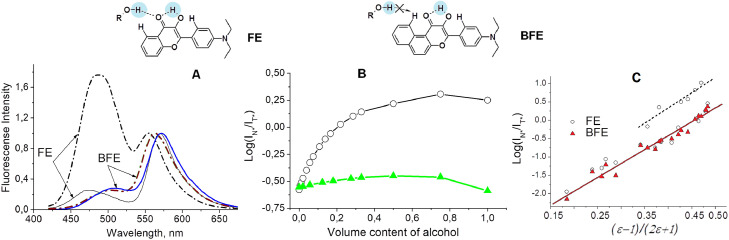
Structures of FE and BFE dyes showing their abilities to impede H-bonding with protic solvent. A) Fluorescence spectra of FE and BFE dyes in ethyl acetate (solid lines) and 2-methylbutanol-2 (dash-dot lines), the solvents of an equal dielectric constant. B) Logarithm of fluorescence intensity ratio I_N*_/I_T*_ as a function of volume content of 2-methylbutanol-2 in ethyl acetate for FE (black) and BFE (green) dyes. C) Logarithm of the fluorescence intensity ratio I_N*_/I_T*_ versus solvent polarity function *f(ε)* for FE (○) and BFE (▲) dyes. Data from [39].

Sensing the physical polarity of proton media, which is expressed as a function of dielectric constant *f(ε)=(ε−1)/(2ε+1)* using a fluorescent probe, was problematic, since majority of known solvatofluorochromic probes possessed an H-bond accepting group, so they has an additional sensitivity to the H-bonding molecules. On this reason, the existing “empirical polarity” scales [38] simultaneously characterize the cumulative effect of the properties of the solutions, namely, the polarity and the H-bonding ability. A successful attempt to eliminate the probe spectral sensitivity to H-bonding was achieved by the synthesis of the benzo-analogue of 3-hydroxyflavone BFE [39] (Fig. 6). The BFE is still the only probe sensitive only to electric fields in solutions, not sensitive to H-bonding, thereby pushing the term "empirical polarity" into the past.

Elimination of the sensitivity of the BFE probe to hydrogen bonding was achieved by spatial shielding of the oxygen of the C=O group, the only atom in BFE capable to form hydrogen bonds in the excited state of the probe. Spectral and chromatographic data confirmed that in the BFE molecule, the hydrogen atom of benzene ring and the 3-OH group prevent the formation of intermolecular H-bonds by the C=O group. The oxygen atom of the 3-OH group is also blocked by lateral phenyl from intermolecular H-bonding. Steady-state fluorescence of BFE probe shows the sensitivity to dielectric properties of the solution only and almost no sensitivity to presence of H-bonding molecules (Fig. 6). For example, the BFE probe in ethyl acetate and 2-methylbutanol-2, the solvents of equal dielectric constant shows a similar bands intensity ratio parameter I_N*_/I_T*_ equal to 0.25 and 0.28, respectively. Meantime, the parent dye FE has an increased value of intensity ratio I_N*_/I_T*_ parameter in protic solvent: 1.77 against 0.26 in ethyl acetate (Fig. 6A). Insensitivity of BFE to H-bond donors also takes place in solvent mixtures (Fig. 6B). The spectral parameter Log(I_N*_/I_T*_) shows a quite linear dependence versus physical polarity function *(ε-1)/(2ε+1)* for BFE, while for FE two separate curves for H-bonding and aprotic media are observed. Thus, BFE offers a unique opportunity to measure physical polarity in site of localization expressed as a dielectric constant function of its molecular environment, including aqueous solutions of lipids or proteins.

### Quantification of local water concentration of probe environment in peptide-peptide and peptide-nucleic acid complexes

Water is one of the strongest H-bond donor molecules. It can form two H-bonds as a donor, and can accept two more H-bonds through oxygen lone pairs. These properties can be used for detection of water by fluorescent probe. On practice water detection and measurements of the local water concentration are important in the case of environments with low and middle hydration, such as individual sites of proteins, inside and around DNA/RNA, protein-DNA/RNA complexes, lipid membranes, and micelles. Resolving a problem, it is important to suppress or separate the signal from the probe generated by the polarity of the surrounding molecules. For lipid membranes such task was solved using fluorescent probes on 3HF core by deconvolution of N* band on two components of free probe and its H-bonded complex [40], [41]. However, the developed probes are suitable only for evaluation of low concentrations of water.

**Fig. 7:**
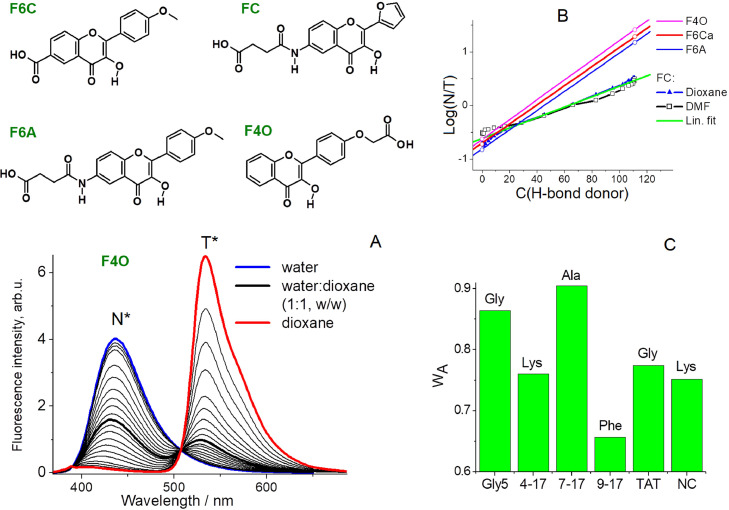
A) Structures of the applied probes and fluorescence spectra of F4O probe in water-dioxane binary mixture of varying compositions. Probe concentration is 3 µM. B) Dependence of Log(I_N*_/I_T*_) versus molar concentration of hydrogen bond donor in water-solvent mixtures for FC probe and linear fits for all probes [43]. C) Dependence of the water access on the nature of peptide and N-terminal amino acid. The W_A_ coefficients are given for FC-labeled (Gly)_5_, peptide (4–17), peptide (7–17), peptide (9–17), Tat(44–61) and NC peptide. The nature of the N-terminal amino acid is indicated over the bars.

The mentioned method was improved by selecting 3HF probes less sensitive to electric fields, with better separation of the fluorescence bands of N* and T* forms [43]. It was shown that the intensity ratio I_N*_/I_T*_ of the probe fluorescence is linearly dependent on the molar water concentration in water - organic solvent mixtures, in a range 10 – 55 mol *L* ^−^ ^1^ of water (Fig. 7). The deviations from linearity were in cases, when preferential solvation of the probe molecule by water or solvent took place. The deviations present additional approvement of correct measurement of local water concentration. Assuming that the mechanism of 3HF response to water molecules is the same when the probe is bound to biomolecules than when it is free, the probe response will result from the interaction of the 3HF atoms involved in ESIPT with water molecules, as it is depicted in [43]. Through H-bonding occurring via the 4-carbonyl group of 3HF, water weakens the intramolecular H-bond and thus slows down or even blocks the intramolecular proton transfer.

This method was applied for measuring hydration of N-terminal labeled peptides and their complexes with oligodeoxynucleotides (ODNs), more particularly to labeled-Tat(44–61) peptide (Fig. 7A). Local concentration of water at the label surrounding was determined for the label alone, in the labeled peptide alone and for labeled peptide complexes with single-stranded and double-stranded ODNs. The level of hydration was then converted into the water access coefficient W_A:_

$$W_{A}={\left[H_{2}O\right]}_{L}/55.5,$$

where 55.5 corresponds to the molar concentration of neat water. W_A_ coefficient appears more adequate for describing hydration level at nanometric scale.

Interestingly, the W_A_ value for the F4O label in the peptide-DNA complex is almost zero, suggesting that in this case the probe has no access to water, which could be explained by a deeper intercalation of F4O molecule into the base pairs [43]. Finally, the hydration of FC probe located at the N-terminus of different peptides was determined (Fig. 7C). It was found that water access mainly depends on the nature of the neighboring amino acid residue, but not on the length of peptide, since shorter peptides with a lipophilic residue at the N-terminus gave lower hydration of the label (compare the data for 4–17 and 9–17 peptides, where the lipophilic phenylalanine strongly decreases the W_A_ value). Besides the N-terminal, other amino acid residues had lower influence on the W_A_ parameter. Thus, the elaborated method of quantitative evaluation of the hydration of multi-channel fluorescent probe gives better insights for the knowledge of structure of biopolymer at binding site of the probe. This result was obtained by reducing the sensitivity of the probe to polarity parameter in the fluorescence intensity ratio channel I_N*_/I_T*_. How to turn off the sensitivity of the probe to hydrogen bonding along the polarity and leave the sensitivity to hydrogen bond acceptors will be shown in the next section.

### Sensing the H-bond accepting ability of a solution

**Fig. 8:**
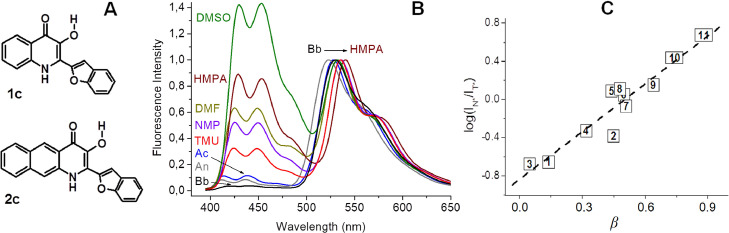
A) Structures of dyes 1c, 2c. B) Normalized fluorescence emission spectra of 1c in aprotic solvents. Abbreviations: DMSO – dimethylsulfoxide, HMPA – hexamethylphosphotriamide, DMF – dimethylformamide, NMP - N-methylpyrrolidone, TMU - tetramethylurea, Ac – acetone, An – acetonitrile, Bb – bromobenzene. C) Dependence of log(I_N*_/I_T*_) on the Abraham's hydrogen bond acceptor basicity parameter for the probe 2c. Numbering of solvents: 1 – toluene, 2 – ethyl acetate, 3 – dichloromethane, 4 – acetonitrile, 5 – methanol, 6 – ethanol, 7 – n-butanol, 8 – dioxane, 9 – THF, 10 – DMF and 11 – DMSO. C) Log(I_N*_/I_T*_) parameter of dye 2c fluorescence Abraham's basicity parameter *β.*

Electric fields, H-bond donating and accepting ability are the driving forces that always work in biosystems at neutral pH. To complete the set of probes, we elaborated first fluorescent probes for the measurement of local H-bond accepting ability in solutions. In such probes the sensitivity to polarity and H-bond donating ability should be suppressed. Convenient scaffolds for their development are 3-hydroxyquinolones (3HQs), in which the transition dipole moment is oriented across the length of molecule, as evidenced by quantum chemistry calculations and their low molar extinction coefficients. After several attempts, positive results were achieved by the synthesis of 2-benzofuryl derivatives of 3HQs (Fig. 8) [44,45].

It was found, intensity ratio I_N*_/I_T*_ of the probe fluorescence is in linear dependence with the Abraham's basicity parameter *β* of a solvent. A set of parameters composes the most popular scale of H-bond accepting ability (Fig. 8C) [46]. What is important this dependence is common for protic or aprotic solvents that open a possibilities for applications of these probes for study bioobjects. The best refinement from the interference of polarity was achieved for the probe 2c, for which is obtained the best fit with the basicity parameter of the solvent (Fig. 8C, [45]). A mechanism of inhibition of ESIPT reaction in 3HQs by basic solvent was also proposed [44].

So, in such case the researcher can ignore the information from the channel of total fluorescence intensity, where the local viscosity and H-bond donor ability of the medium are reproduced, to ignore the position of band's maxima, where the polarity is reproduced, and take into account only one useful parameter I_N*_/I_T*_. The chemical structure of the probe provides this possibilities.

## Section 3. esipt probes in detection of cations

### A wide-range pH indicator

**Fig. 9:**
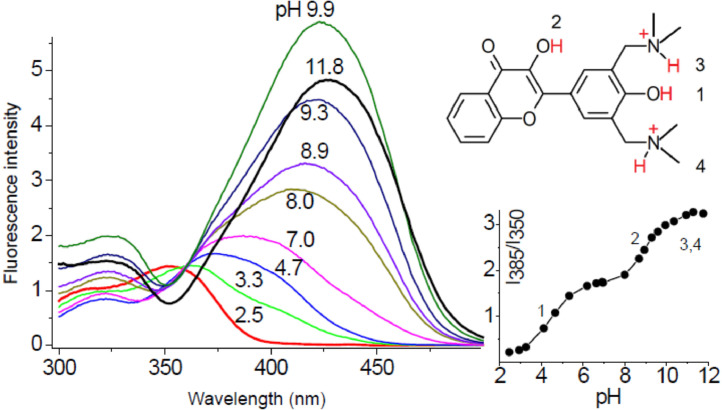
Excitation spectra of FAM345 in the range of pH from 2.5 to 11.8. Fluorescence intensity ratios (in the base 10 logarithm) *I*_385_/*I*_350_ versus pH [47].

The functioning of biosystems takes place within pH range from 2 to 8.5, which exceeds the theoretical operating limits of 4 pH units of the fluorescent pH indicator. In the case of chemical objects, the range of pH changes can be wider. In such cases of wide-range pH measurements, for the ratiometric probe is necessary in some way to expand the range of pH-sensitivity, and to diminish the sensitivity to polarity and H-bond donor property of surroundings. Or to shift this sensitivities to other information channels, so that there would no interference with pH measurement take place. As it was shown above, an ability to sense two sequential interactions appears when the probe possesses two receptors of different affinity to analyte [30]. This idea was applied in the design of a wide-range pH indicator FAM345 (Fig. 9, [47]). To shift the deprotonation event to lower pH values, basic amino groups were introduced in close proximity to one of the phenolic residues. A similar design approach for a wide-range pH indicator was successfully applied in another work, but based on a structurally rigid pyridine derivative [48].

The FAM345 indicator, showing dramatic changes in both excitation and emission spectra enables to determine a pH index of solutions in the range from 3 to 12 either by intensiometric or by ratiometric methods using fluorescence intensity at one or two wavelengths (Fig. 9).

### Recognition of binding of Mg^2+^, Ba^2+^ and *H*^+^ cations by the 3-hydroxyflavone probe

**Fig. 10:**
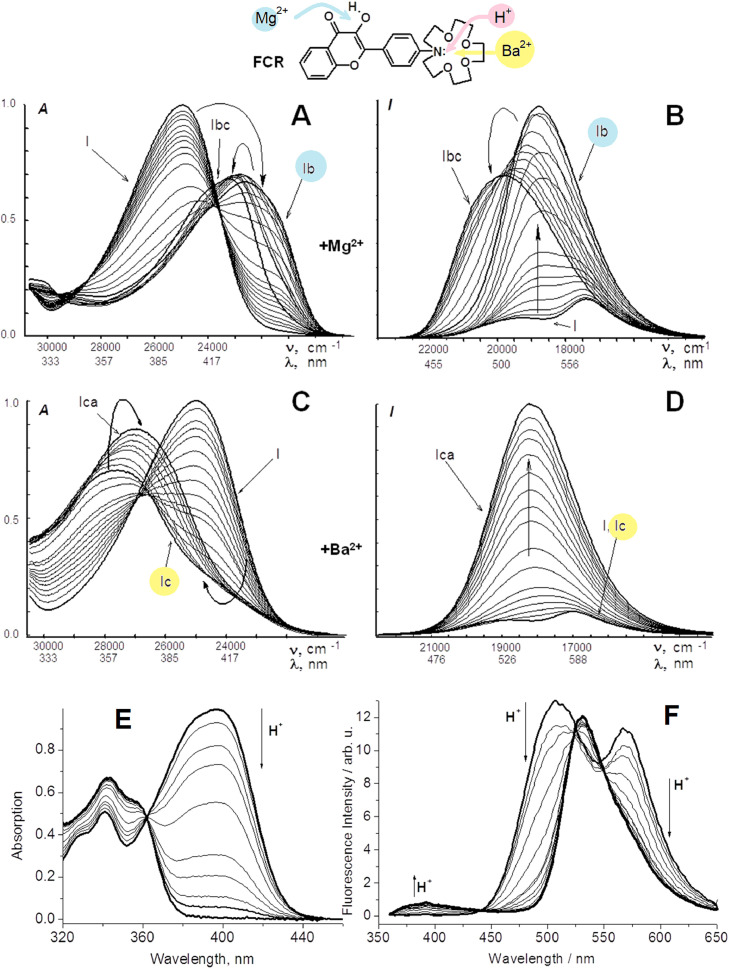
Evolution of absorption (A,C,E) and emission spectra (B,DF) of FCR dye in acetonitrile at changes of Mg^2+^ (A, B), Ba^2+^ (C,D) and *H*^+^ (E,F) concentration [49,51].

3HFs can be considered as ion chelators since their proton transfer system is able to bind metal polyvalent cations. As in the case of pH-indicator above, the installation of an additional ion receptor in an opposite site of the molecular dipole can give an opportunity to recognize the cation by the dye fluorescence response profile. This idea was tested with a 4′-aza-15-crown (FCR) derivative of 3HF in order to detect Mg^2+^ and Ba^2+^ in dry acetonitrile (Fig. 10) [49,50].

FCR probe shows different spectral behavior in presence of Mg^2+^ and Ba^2+^cations. A new bathochromic band corresponding to complex 1b appears in the absorption spectrum when Mg^2+^concentration increases. This band is progressively undergoing a blue shift when the complex 1bc of stoichiometry 1:2 is forming. These variations can be explained by the sequential steps, where the first Mg^2+^ cation is bound by the hydroxy‑carbonyl chelator, in place of a proton, and the second one – by the macrocycle. Time-resolved spectroscopy of complex 1bc shows its partial ejection of Mg^2+^ from the crown-cycle in the excited state as a result of electron charge transfer from the nitrogen atom to the conjugated system (Fig. 10 [50]).

In case of Ba^2+^concentration increase, an initial blue shift of the absorption spectrum is observed corresponding to the formation of the complex 1c by the crown residue. The emission spectrum at such levels of Ba^2+^concentration is unvarying, pointing out the complex dissociation in S_1_ state. At further increase of Ba^2+^concentration, a gradual red shift is observed in absorption, showing the formation of the complex 1ca, for which Ba^2+^is lost by the crown-cycle in S_1_ state, in agreement with the modifications observed for the emission spectrum. A clear confirmation of Ba^2+^ ejection in S_1_ state was obtained by time-resolved spectroscopy [50]. Further experiments were showing a similar behavior with Ca^2+^ in acetonitrile [52].

The protonation of FCR in acetonitrile proceeds through the nitrogen atom and results in the formation of a new band which is strongly blue-shifted [51]. A more efficient formation of the tautomer form was observed in the excited state for the protonated molecule, due to which both its absorption and emission bands are very close by their shape and position to the unsubstituted 3HF ones (Fig. 10F). Similar spectral changes were observed in water-ethanol solution for this probe [52]. Thus, FCR probe by its steady-state spectra can differentiate both in absorption and emission the binding with Mg^2+^, Ba^2+^and *H*^+^ions. These results demonstrate the possibility of recognition of cations of different radii by multi-channel fluorescent probe. An example of Ca^2+^ cation recognition by 3HF probes was also published [52].

## Section 4. biomolecules and biomolecular complexes study with the esipt probes

### Applications of 3HF probes to the studies of micelles and lipid membranes

Besides the bands of N* and Т* forms, ones of their H-bonded species N*-Н and T*-Н, in fluorescence excitation and emission spectra were registered the bands of protonated and deprotonated species of 3HFs, the last one in pH region higher 7.0. A significant difference in the position and intensity of the mentioned bands showed broad prospects for the application of 3HF in studying the properties of solutions, including microheterogeneous ones. Because in such case applying a detailed analysis of the spectral curves the researcher is able to obtain a wider set of information about the nature of the probe's surrounding. Applications of 3HFs as multi-channel probes in investigations of micelles [53], [54], [55] and lipid membranes [56], [57], [58], [59] started three decades ago simultaneously with studies on proteins [36,37]. Initially, five fluorescence parameters were used to characterize the probe environment, namely fluorescence quantum yield, intensities and positions of N* and T* bands. The parent 3HF was shown to correctly report the formation of micelles in water [53] simply by the increase of fluorescence quantum yield, while the N*/T* intensity ratio was considerably reduced confirming the decrease of polarity in a micellar environment [53], [54], [55]. Dialkylamino derivatives like FE, FME and FCR probes were shown to be more sensitive and thus more informative than parent 3HF [55].

**Fig. 11:**
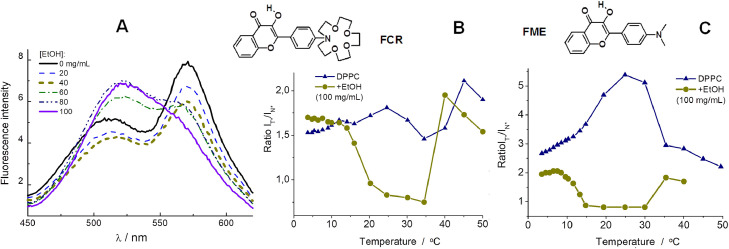
(A): Fluorescence spectra of FCR dye in DPPC multi-lamellar vesicles with increasing concentrations of ethanol. (B): Changes of FCR I_N*_/I_T*_ intensity ratio with increasing temperature. (C): The same parameter for FME probe in DPPC vesicles without and with 100 mg/mL of ethanol [56].

The probes FCR and FME provided new information about temperature-induced transitions and small additives effects on lipid membranes [56]. For example, increase of ethanol concentration in dipalmytoylphosphatidylcholine (DPPC) multi-lamellar vesicles induced the interdigitation of the membrane bilayer as shown by the increase of the ratio I_N*_/I_T*_, suggesting increase of H-bonding specie content around the probe molecule (Fig. 11A). The same parameter was also dramatically modified after addition of cholesterol or temperature increase, showing the changes of hydration of probe surrounding. (Fig. 11B,C). The wider range effect observed with FME was suggested as relocalization of the probe within the bilayer, that confirmed by fluorescence quantum yield changes in mentioned experiments and special time-resolved measurements [57]. Thus, in experiments with lipid membranes, information about the hydration of surrounding can be obtained through the channel of intensities’ ratio I_N*_/I_T*_, and about the viscosity – through the channel of total fluorescence intensity.

### Study of a peculiar effect at a water-lipid interface

FME anion formation was registered at pH values in the range 6.7–7.6 in presence of N-(2-hydroxyethyl)piperasine-N’-ethylsulfonate (HEPES) buffer when labeling anionic egg yolk phosphatidylglycerol (EYPG) vesicles [60]. The anionic form of FME appeared as a shoulder on the red edge of both absorption and excitation spectra of the probe while no anionic form was oberved in neat buffer or in EYPG vesicles prepared in phosphate buffer at same pH values. It should be stressed that the anionic form of FME probe earlier was registered at much higher pH values.

The mentioned above result was achieved due to the possibility of comparing the fluorescence intensities of the anionic form (460 nm) and the normal form (400 nm) in the excitation spectra. The shift of the band maximum of the anionic form to 460 nm in the lipid membrane compared to its position in water (430 nm) indicated its additional stabilization in the electrostatic field. Therefore, the use of three information channels of the fluorescence intensities at 400, 430 and 460 nm in the excitation spectrum and the analysis of the band shapes of the emission spectra made it possible to establish the presence of unique molecular complexes on the surface of the lipid membrane.

The detailed study of HEPES-EYPG vesicles system by steady-state and time-resolved fluorescence spectroscopy allowed explaining this unusual phenomenon. It was suggested that the FME anion formation is a result of probe localization in the electrostatic field of the triple charged layer formed by EYPG-HEPES aggregates. The entering of FME probe into this layer is promoted by H-bond formation of the 3-OH group with basic nitrogen atom of HEPES molecule. In the absence of HEPES such an effect was not observed. The evidence of FME anion location in the electrostatic field is that the position of its maximum in the absorption and excitation spectra was anomalously shifted to the red in comparison with one in water solution. Thus, the specific structure of EYPG-HEPES surface results in partial dissociation of FME probe even at neutral pH values of bulk solution.

### Labeling of peptides to study peptide-lipid membrane and peptide-peptide interactions

Environment-sensitive labels present a powerful tool in the study of interaction of peptides with lipid membranes. The multi-channel fluorescent probes based on 3-hydroxyflavone fluorophore give the most detailed information about peptide surroundings in such experiments, as the analysis of their spectral parameters can differentiate the dielectric interactions from H-bonding ones [39], and to measure the level of hydration of different areas of lipid membrane [61].

**Fig. 12:**
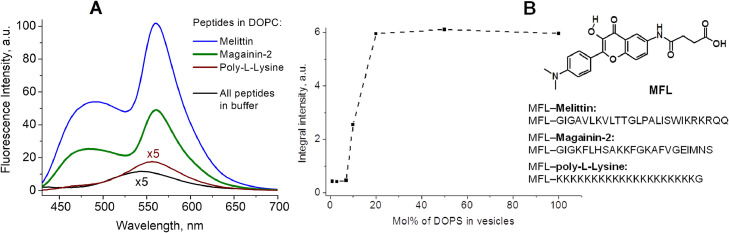
A) Fluorescence modifications on binding of MFL-labeled peptides to DOPC vesicles. A): Emission spectra of the peptides in absence (dash) and in presence of LUVs composed of neutral DOPC lipids. B): Dependence of poly-l-lysine/LUV binding versus the percentage of negatively charged DOPS lipid in DOPC-DOPS vesicles. Structure of MFL label and amino acid sequences of studied peptides are presented on the right side of the figure. Concentration of peptide and lipids was 0.3 and 100 μM, respectively. Applied buffer: 20 mM phosphate, 150 mM NaCl, pH=7.4. Data are taken from ref. [62].

To study the membrane binding of three different peptides, melittin, magainin and poly-l-lysine, they were covalently labeled at their N-terminus with 3HF probe [62]. In this work, three information channels were used to fix the binding of peptide to the membrane and to determine the parameters of hydration and polarity of the environment: the total fluorescence intensity, the ratio of the intensities of the H-bonded H—N* and normal N* forms, I_NH*_/I_N*_, as well as the position of the maximum of the N* band. The binding can be easily followed by the increase of fluorescence intensity (Fig. 12A,B). Melittin and magainin bind to zwitterionic lipid bilayers, while poly-l-lysine binds only to negatively charged lipid bilayers. The deconvolution of fluorescence spectra into three components of H—N*, N* and T* bands demonstrates that the hydration at the N-terminus is approximately the same for all peptides in all tested membranes, while the polarity differs. The highest value of environment polarity was observed for poly-l-lysine in DOPC/DOPS (8:2) lipid vesicles and the lowest one for melittin in DOPC vesicles. A lower polarity means a deeper penetration of the peptide N-terminus into the lipid bilayer. This was confirmed by parallax quenching experiments using different location quenchers. The orientation of the label in giant unilamellar vesicles was estimated by fluorescence anisotropy of N* and T* forms emission in fluorescence microscopy experiments. It was evidenced that poly-l-lysine displays an orientation of the label orthogonal to the membrane surface, while melittin displays both parallel and orthogonal orientations. The work [62] represents a good example where a single multi-channel probe simultaneously gave three different informations: binding, location and orientation of a peptide domain, which would not be possible with just intensiometric probes. This strategy of peptide labeling by the N-terminus with a multi-channel probe was created [63] and then improved for labeling by cysteine residue [64]. The method was successfully applied to study peptide-lipid membrane interactions and α-synuclein protein aggregation processes [65,66] .

### Fluorescent α-l-amino acid for the study of peptide interactions with lipids

**Fig. 13:**
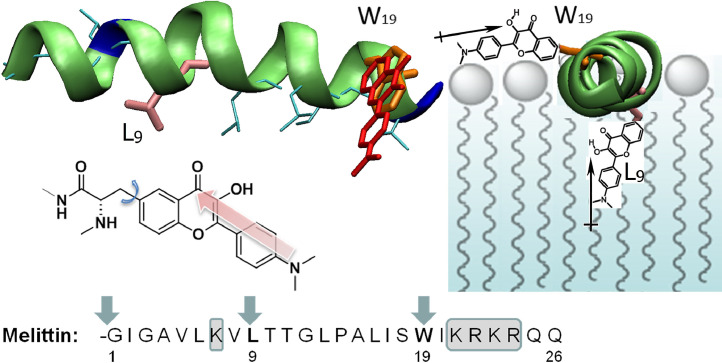
Spatial structure of melittin showing the orientations of AFaa fluorophore depending on the positions of substituted aminoacids. Five unstructured residues at basic C-terminus are not shown. Middle: the structure of AFaa amino acid. Bottom: sequence and labeling scheme of melittin. The alternative directions of the transition dipole moments of the fluorophore are shown by arrows. Cationic amino acids of melittin are marked in gray.

An improvement of the method presented before [62] consists in the synthesis of a fluorescent α-l-amino acid AFaa containing a 3HF fluorophore, which makes it possible to set the polarity and hydration of environment around a certain segment of the peptide. Such an exotic amino acid was introduced in two melittin peptide variants by solid phase synthesis instead of leucine-9 (L9) or tryptophan-19 (W19, Fig. 13). Melittin, a 26 amino acid, cationic peptide from honey bee was chosen as a membrane-binding peptide whose properties are well-studied by various techniques, including fluorescence quenching, оriented CD, X-ray, neutron scattering and MD simulations methods [67], [68], [69], [70]. In contrast to covalent labeling approaches via a flexible linker, there the fluorophore is closely attached to the peptide backbone in desirable position, with a well-defined orientation. This enabled a more precise determination of the peptide insertion and orientation in lipid membranes. The peptide labeled at the N-terminus by the MFL probe was kept for comparison [71]. So, three information channels were used to fix the binding of peptide to the membrane and to determine the parameters of hydration and polarity of the environment: the total fluorescence intensity, the ratio of the intensities of the H-bonded H—N* and normal N* forms, I_NH*_/I_N*_, as well as the position of the maximum of the N* band.

Due to high sensitivity of AFaa emission towards the hydration of environment, it was possible to register the kinetics of melittin tetramer dissociation after dilution of the solution. The dissociation was registered by the fluorescence intensity decrease. Considering the N*/T* intensities ratio, it was concluded that the lowest hydration corresponds to the L9 position of melittin tetramer, while in monomer, melittin exhibits approximately the same hydration for all studied sites. A strong electrochromic effect, expressed by a blue shift of the N* band emission, was observed for the label at position W19 due to the electrostatic influence of the neighboring cationic amino acid residues both in the tetramer and the monomer.

The insertion of the labeled peptide into the membrane bilayer of DOPC large unilamellar vesicles resulted in a strong (up to 20-fold) increase in the fluorescence emission intensity and in the appearance of two emission bands. Such an observation is usual for 3HF probes embedded within the viscous hydrophobic lipid core of a bilayer. The label at position L9 shows the lowest I_N*_/I_T*_ intensity ratio corresponding to the deepest insertion of the peptide segment into the membrane. For the label at position W19, the electrochromic influence of neighboring cationic residues disappears, showing that the lipid environment induced a clear separation between the charged peptide residues and the probe. The emission spectrum observed in living cells was quite similar to that ones observed in DOPC vesicles, which proves that the lipid environment of melittin in model bilayers and in cell membranes does not greatly differ.

Also should be stressed, the macrodipole of the alpha helix is poorly interferes with the charge transfer and ESIPT of the probe, since it is close to the orthogonal orientation relatively to the probe molecular dipole. Second, and the main issue, the probe dipole in any of orientations is surrounded by lipid molecules in membrane or by water in aqueous solutions. So, the charges of phosphocholine residue and the dipoles of fatty acid ester groups or water give main influence on the fluorescence of the AFaa probe.

Fluorescence microscopy experiments with giant vesicles and live cells using a polarized light excitation show drastic differences in the orientation of AFaa label at L9 and W19 positions. This means that in cell membranes, as in lipid bilayers, melittin helix is preferentially oriented parallel to the membrane surface. Thus, the effective label construction and the combined utilization of several fluorescence parameters enabled to simultaneously obtain new and unique data about both peptide location and orientation in lipid membranes.

### High-selectivity detection of adenosine triphosphate (ATP) anion

**Fig. 14:**
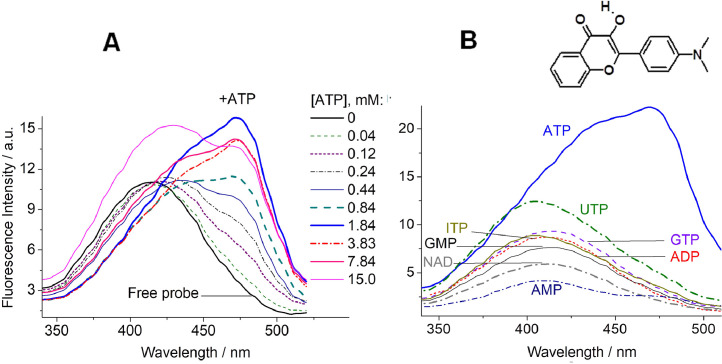
Fluorescence excitation (A) spectra of FME probe in aqueous solution of sucrose and HEPES-carbonate buffer with increasing ATP concentration. Emission wavelength: 554 nm for all excitation spectra. Concentrations are: 250 mM (sucrose), 10 mM (HEPES), 40 mM (carbonate); pH 7.4. C) Fluorescence excitation spectra of FME probe in the presence of nucleoside phosphates. Data are taken from [72].

A highly selective detection method of ATP anion in water was obtained by using the 4′-dimethylamino-3-hydroxyflavone (FME probe, Fig. 14A) probe [72,73]. The interaction with ATP in an aqueous medium induces a new band in the fluorescence excitation spectrum of FME, having a large red shift. Such a shift can only be assigned to the appearance of anionic form of FME in a complex with ATP, where FME is stabilized by the electric field of the tetra-charged ATP anion. In this case, as in the case of the anionic form of the probe in lipid membranes (see Section 10), the electrostatic effect from the environment was registered. At the same time, the fluorescence intensity increases with ATP concentration (Fig. 14B), which is, in addition to the electrostatic effect, the result of an increase in viscosity and a decrease in the concentration of water around the probe. In this experiment, binding with an organic molecule was registered by the channel of the total fluorescence intensity. The unusual effect of a new band formation in the excitation spectrum created the possibility to register the electrostatic effect and, following it, the concentration of the negatively charged analyte in the solution. In such an experiment, up to five channels of information transmission from the object were used. Most surprising was the unusually high selectivity of FME probe in respect to ATP as compared to the other nucleotides (Fig. 14B). The other nucleotide anions also induce some increase of FME fluorescence intensity, but in a much lesser extent than ATP anion and without red-shifted band.

The formation of two complexes was registered by the analysis of spectrophotometric titration data. First, a higher affinity FME-ATP complex of 1:1 stoichiometry leads to a strong hypochromicity of the absorption spectrum of the FME probe. A lower affinity (ATP)_2_-FME complex results in a strong increase of the fluorescence intensity (up to 20-fold), due mainly to the appearance of FME anionic form, as shown by the important red-shift (60 nm, Fig. 14A,B) in both excitation and emission spectra. Using this strong enhancement of fluorescence intensity in the presence of ATP, FME was used successfully to monitor the succinate-induced production of endogeneous ATP in mitochondria. This was confirmed by the huge shift observed in FME fluorescence excitation spectra following the addition of succinate [73]. As a consequence, the multi-channel probe FME, through its interaction with ATP molecule, allows the selective detection of ATP anion and thus can be considered as a starting molecule to design even more efficient ATP sensors.

In furtherance of these studies, an uracil-linked 3-hydroxyflavone [74] and 33 probes from 3-hydroxyflavone and 3-hydroxyquinolone series have been shown their good multi-channel response in fluorescence excitation and emission modes to the presence of ATP [75]. It was found that 30 compounds from the set give a new band in the spectrum in the presence of ATP in the concentration range from 2∙10^−6^ to 5∙10^−2^ mol *L* ^−^ ^1^. The increased range of ATP detection was explained by the formation of two consecutive compleaxes probe-ATP with stoichiometry 1:1 and 1:2. The last work has shown that the 3-hydroxyflavone core is the scaffold that binds to ATP within its biological concentrations, and the substituents only partially change the affinity of the probe to ATP.

### Recognition of peptide-nucleic acids interactions

The productive studies of lipid membranes with 3HF probes have stimulated the development of the labels for the study of peptide-nucleic acid interactions. To this aim, the peptide corresponding to the zinc finger domain of the human immunodeficiency virus HIV-1 nucleocapsid protein was synthesized, labeled by the N-terminus by 3HF probe and successfully applied to study the interaction with oligodeoxynucleotides (ODNs) and DNAs [41]. To improve the approach, several 3HF labels were covalently attached to the N-terminus of the Tat(44–61) peptide of HIV-1 virus [42]. Three new labels contained the linkers of different length and were attached at different positions of the same fluorophore. These fluorophores from 3HF family provided a higher sensitivity towards hydration of probe surroundings and decreased sensitivity to dielectric interactions.

**Fig. 15:**
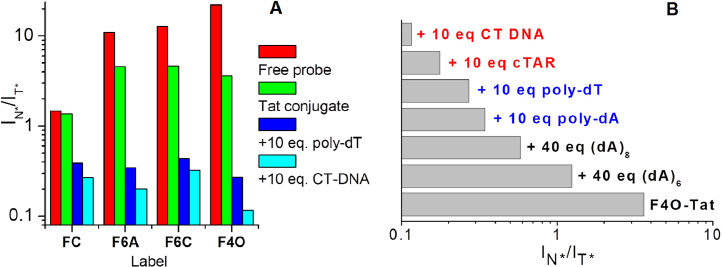
A) N*/T* intensity ratios for free FC, F6A, F6C and F4O labels (dark gray) and for their conjugates with Tat(44–61) peptide in the absence (gray) or in the presence of poly-dT (black) or CT-DNA (light gray). B) Fluorescence ratiometric response of F4O-labeled Tat(44–61) peptide on interaction with various oligonucleotides. Concentration of peptide was 0.5 µM in 10 mM phosphate buffer pH 7.0 and 30 mM NaCl. The ODNs were added at a ratio of 10 or 40 bases or base pairs per peptide molecule. In all cases, the full complexation of the peptide by the ODNs was achieved, as no change in the N*/T* ratio was observed after a two-fold increase in the concentration of ODNs [42].

At first stage spectroscopic properties of labeled peptides were studied in the presence of different ODNs. Their absorption spectra indicated the effective binding with ODN as shown by the decrease of absorbance, but the most distinct sensing was obtained by fluorescence spectroscopy. As compared to FC, the three other probes provided a better response (about 3-fold larger) to the binding, in line with their higher sensitivity to the environment. Indeed, dramatic changes in the I_N*_/I_T*_ ratio were induced by the binding of ODNs of different length, sequence and strandedness, single-stranded (ss) or double-stranded (ds), to the labeled peptides (Figs. 15A). The level of probe hydration was related to its ability to stack with DNA bases. Owing to this consideration, the best resolution was obtained with F4O label, presenting the highest lipophilicity and the most compact geometry. Thus, using the I_N*_/I_T*_ ratio of the F4O label, it was possible to distinguish the interaction of the peptide with four types of DNAs, namely small ss ODNs, ss DNAs, partially ds DNAs and fully ds DNAs (Fig. 15B). As a consequence of the high sensitivity of the F4O label to the nature of the bound nucleic acid, it was possible to compare the relative affinities of this peptide to ss and ds DNAs. Thus, this new generation of hydration-responsive probes appears as a highly sensitive ratiometric tool to site-selectively monitor the binding of peptides to different types of nucleic acids.

### Characterization of interaction sites in DNA-DNA, dna-rna and peptide-nucleic acid complexes

**Fig. 16:**
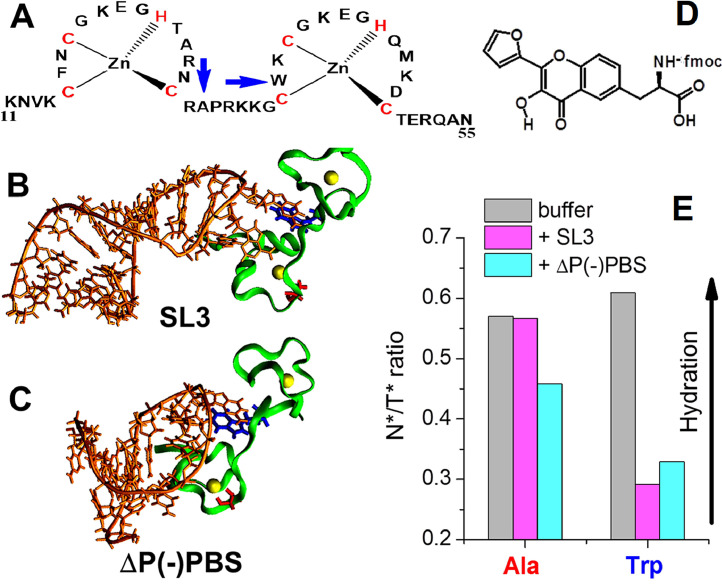
NC(11−55) composition (A). 3D structures of NC complexes with SL3 (B) and ΔP(-)PBS (C) based on NMR data. Only the NC(11−55) part is shown. Zn atoms are represented as spheres. D) The structure of 3HCaa amino acid. E) Fluorescence intensity ratio I_N*_/I_T*_ for NC(11−55)-*A30*–3HCaa and NC(11−55)-*W37*–3HCaa peptides in buffer and bound to SL3, SL2 or ΔP(-)PBS oligonucleotides [76].

Further on, the elaborated method of quantitative evaluation of hydration [43] was applied to the study of separate site contacts of peptide with three different ODNs. For this purpose, a dual-fluorescence *α-l*-amino-acid 3HCaa was synthesized on the basis of 2-furyl-3-hydroxychromone [76]. The amino acid derivative possesses fluorescence properties similar to that of 2-furyl-3-hydroxychromone (FHC) probe (Fig. 16). It was introduced by solid-phase synthesis either in position A30 or W37 of the 11–55 fragment of nucleocapsid protein (NC peptide), a component of human immunodeficiency virus HIV-1, instead of alanine and tryptophan residues, respectively. Then, both peptide variants were tested in binding experiments with the ODN sequences known to have increased affinities for NC(11–55) peptide, namely SL2, SL3 and ΔP(-)PBS.

The 3HCaa label gives different response on binding of the peptide with ODNs, depending whether it is located in A30 or W37 position. The binding of A30 variant with SL2 and SL3 RNAs results only by the T* band red shift, while its binding with ΔP(-)PBS sequence additionally results in the decrease of the intensity ratio I_N*_/I_T*_, showing a stronger contact of A30 site of peptide with ΔP(-)PBS. In the case of W37 variant, on interaction with ODNs SL2, SL3 and ΔP(-)PBS, a strong change in the fluorescence spectrum was observed, with notably a large drop in the ratio I_N*_/I_T*_, which can be assigned to a decrease of the local hydration of the probe environment caused by the close distance to RNA segment . These results can be rationalized by a larger distance between A30 residue and the nucleic bases within the complexes an consequently a higher exposition to water. These data correlate well with the NMR data for the NC(11–55) peptide and for the whole NC sequence (Fig. 16B,C).

In both cases, the water access W_A_ parameter can be calculated from the calibration procedure [43] for the interaction sites of A30 and W37 residues with ODNs. W_A_ parameter drops to 0.32–0.41 for W37 variant, but only to 0.49 −0.54 for the A30 variant, to be compared to 0.57 for the free labels.

Thus, for the first time, a fluorescent l-amino acid based on a 3HC probe, incorporated at different positions by peptide synthesis into a peptide, opens the possibility not only to quantitatively characterize the interaction of the peptide segment with other peptide parts or with nucleic acids, but also to determine the level of water access at the interaction site. Such higher informational content directly results from a clever utilization of this family of multi-channel probes, as compared to more classical probes (see [76]).

**Fig. 17:**
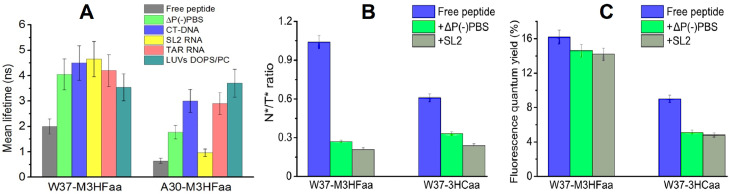
Fluorescence lifetime imaging data of solutions of W37-M3HFaa and A30-M3HFaa, either as free peptides or in complexes with ODNs or LUVs Comparison of N*/T* ratio (B) and fluorescence quantum yield (C) values for the NC(11–55) peptides labelled at position 37 by M3HFaa or 3HCaa.

In the following work a new *α-l*-amino acid bearing the 4′‑methoxy-3-hydroxyflavone fluorophore (M3HFaa) was synthesized, incorporated into NC(11–55) peptide at the same A and W sites and studied in an experiment on binding with oligodeoxynucleotides (ODNs) and lipid vesicles (LUVs) [77]. The dual emission of M3HFaa was found to be substantially more sensitive to hydration as compared to 3HCaa (Fig. 17). It was shown that M3HFaa amino acid does not change the spatial structure and functions of the peptide. Binding of the labeled peptides with nucleic acids and lipid vesicles produced a strong switch in their dual emission, explained as the strong decrease of local hydration. This switch was also associated with the appearance of long-lived fluorescence of the T* form, as a consequence of the rigid environment in the complexes that restricted the relative motions of the M3HFaa aromatic moieties. The strongest restriction and thus the longest fluorescence lifetimes were observed at position 37 in complexes with nucleic acids, where the probe likely stacks with the nucleobases. Based on the dependence of the lifetime values on the nature of the ligand and the labeled position, two-photon fluorescence lifetime imaging was applied to identify the binding partners of the labelled peptides microinjected into cells. Thus, M3HFaa appears as the most sensitive tool for monitoring site-selectively peptide interactions in solution and living cells.

The work on study of the single-stranded and double-stranded ODN complexes [78] should be considered as an extension of the hydration evaluation method to DNA and RNA species labeling. In the mentioned work the fluorescent deoxyribonucleotide with uridine-3-hydroxychromone base was synthesised and installed in the series of ODNs. Since the fluorescent base almost did not disturb the spatial structure of DNA/DNA and DNA/RNA duplexes, for the first time the local water concentration in different sites around the complexes was estimated. Basing on band intensity ratio N*/T* and on the position of T* band, the method provided the highest sensitivity and allowed discriminating between matched and mismatched dsDNA, as well as between B- and A-forms of DNA/DNA and DNA/RNA duplexes. Detailed review on design and application of nucleotides possessing multi-color fluorescence presented in [79].

### Fine labeling of small antimicrobial cyclic peptide

Labeling biomolecules with fluorescent probes is an established tool for biophysical studies. However, it remains underused for small peptides, as there is a serious threat in such case to change affinity and selectivity of the peptide interaction with its target in the object. In a recent work, the fluorescent amino acid probe 3HCaa was incorporated into a known antimicrobial peptide, cyclo[RRRWFW] (cWFW), in place of aromatic residues [80]. It was shown that the installation of the probe at the W_1_ site does not significantly reduce the antibacterial activity, although it slightly changes the conformation of the peptide in model solutions. The equilibrium between two conformations of the peptide in lipid membranes, micelles, trifluoroethanol and buffer solution was suggested by the data on the position and intensity of bands in the fluorescence and absorption spectra. In this work the maximal number of information channels created by the probe in the steady-state spectra was applied. The shape and band maximum position in the fluorescence and absorption spectra were analyzed. For example, for all tested peptide variants due to the presence of three cationic groups of arginine, a significant difference in the conformations of the peptide was registered according to the position of the maximum in absorption and fluorescence excitation spectra obtained in lipid membranes (360 nm), water (370 nm), ethanol (440 nm) and dimethylformamide (450 nm).

Comparing the results obtained with other probes on cWFW peptide modification, 3HCaa can be considered the first adequate label for the study of small peptides. This feature allowed to get insights into conformational equilibria of the labeled peptides localized in cells, and assess the polarity and hydration of the local environment around the label by confocal fluorescence microscopy. It was found that the labeled peptides efficiently penetrated cancerous cells and localized mainly in lipid-containing and/or other nonpolar subcellular compartments. In the zebrafish embryo, the peptides remained in the bloodstream upon injection into the cardinal vein, presumably adhering to lipoproteins and/or microvesicles. They did not diffuse into any tissue to a significant extent during the first 3 h after administration. This study demonstrated the advantages of fluorescent labeling by multi-channel probes to elucidate the sites and mechanism of action of peptides in organism.

### Before conclusions: how the parameters of probe fluorescence correspond with channels of information transmitting

Fluorescence of a dye in solution can be too complex a phenomenon to fully interpret the nature of its fluorescence. The Jablonsky diagram (Fig. 2) only partially explains all the complexities. Only in separate cases one parameter of fluorescence (for example, band intensity) conveys information about the change of one parameter of a medium (for example, viscosity). In most cases, the influence of molecules of probe's surrounding is transmitted in several information channels simultaneously: by band intensity, by its position, shape and half-width. In such cases, time-resolved fluorescence spectra carry additional useful information. Frequently, the changes in band position, shape and half-width indicate the changes in molecular environment of the probe in total. Often, such subtle changes are ignored in scientific research on the reason of a small spectral effect or a lack of deep understanding of the observed phenomenon. As a result, in publications only one, the most striking effect from the whole set observed is presented and discussed. So, multi-channel transmission of information can be carried out only by the probes that have the largest amplitude of excited-state effects in their combination: ICT, ESIPT and others. Moreover, in separate channels there may be a sum of mixed information about the parameters of surroundings, which should be separated by some algorithm. A complex situation, which in most cases simplifies by selection of two channels of ratiometry [81] and deserves further attention of scientists in the search for more advanced methods of quantitative information processing. This is a way to progress in fluorescence sensing.

## Conclusions

Two phenomena are frequently used in the design of ratiometric probes with multi-band fluorescence, namely photoinduced intramolecular charge transfer (ICT) and excited state proton transfer (ESIPT). Despite ICT was up to now the most popular one, the introduction of ESIPT, more particularly for 3-hydroxyflavones and 3-hydroxyquinolones probes, is presently becoming very attractive. This is due to additional channels of information created by these probes by changing the intensity and position of each of the bands in the fluorescence excitation and emission spectra. Each channel can be separately tuned either by the structure of the fluorophore or the presence of different analyte receptors and transmit information about several physical parameters of probes location. This strategy led to new achievements, including the measurement of local dielectric constant in the probe environment, local H-bond accepting ability, local concentration of water and concentration of ATP in aqueous solutions, all tasks which were quite difficult to achieve with ordinary intensiometric probes.

In most cases, the main informational channel, which is usually the intensity ratio of the two emission bands is used as the main tool for the observation of the process under study, while the other channels (e.g. band positions, their halfwidth, quantum yield, fluorescence anisotropy, etc.) are more often used to control other possible processes in experiments.

Such multi-channel probes were used to noncovalently label lipid membranes, allowing for example to measure dipole and transmembrane potentials and to detect cell apoptosis. They allow also deciphering the structure of raft membranes and also some particular effects concerning the influence of HEPES buffer molecules on the interfacial behavior of phosphatidylglycerol vesicles.

In the field of cation sensing, such multi-channels probes are able to distinguish between different fluorescence signals upon binding with *H*^+^, Mg^2+^and Ba^2+^ cations, giving the opportunity to selectively recognize these cations of different radii by a single probe. Also these probes allow sensing the pH in a wide range.

Multi-channel probes are effective tools in the study of macromolecule interactions. The most useful peculiarity is that they inform simultaneously about several physical parameters of the environment, such providing a better insight about the studied system. As an example, the total fluorescence intensity of such probes could report about local viscosity, the N*/T* band intensity ratio – about local hydration, and the position of N* band – about local polarity, while the polarization of fluorescence could report more about orientation of the probe or even the whole macromolecule. Labeling of peptides by amino acid-mimicking multi-channel probes, via peptide synthesis, opens a new route to study peptide location, and level of peptide site interaction with other biopolymers (nucleic acids, peptides and proteins). The orientation of an α-helix peptide relatively to a cell membrane surface can be also determined by this way.

Thus, comparing multi-channel probes presented in this review with other more classical fluorescent probes, it can be concluded that they are more informative and more accurate tools.

## Section 5. prospects

Since proton is the lightest nucleus of an atom and its migration in a molecule leads to striking changes in electronic structure together with significant and fastest changes in fluorescence properties of the molecule in time scale, perhaps that is why the phenomenon of ESIPT has become the most convenient in the creation of multi-channel fluorescent probes. A logical step to increase the number of information channels from the probe was the development of dyes with two proton transfer systems, both same in structure or different, located in close proximity or at different poles of the molecule [82]. Even a dye with three ESIPT systems was synthesized [83]. It was established that sensitivity of such systems to general properties of environment increases significantly [84], and implementation of one- or two-proton transfer depends primarily on the symmetry of the molecule, and only on the second turn - on the nature of surroundings [78]. Further progress in this direction requires further efforts in organic synthesis and in the study of fluorescent properties of such structurally complex compounds.

Another approach to increase the number of information channels of the probe was the combination of ESIPT and excitation energy transfer phenomena [85,24]. In such molecular devices consisting of a chromophore and a fluorophore, separated in space by up to 10 nm, it is possible to simultaneously determine the distance between them and the nature of their surroundings [37].

After all, in the design of the probe not only ESIPT can be exploited, but several types of chemical reactions. For example, nucleophilic addition or redox type ones. There are already many examples ratiometric (two-channel) probes of such kind design [4]. The creation of multi-channel probes is also already started [29,86,87]. The advantage of such direction lies in the relative ease of combining in the probe molecule the possibilities of two types of chemical transformation with the formation of different fluorophores. An obvious disadvantage of the direction is the slow kinetics of chemical transformations, which lies in the range of seconds and even hours. Сhemical changes in the structure of fluorophore induced by the intermolecular reactions also strongly limits the lower detection limit of the analyte, which usually does not falls down the nanomoles per liters.

## Declaration of Competing Interest

By this letter I declare that I have no conflict of interest and no competing interests such as those usually defined for manuscripts of articles or others that might be perceived to influence the results and discussion reported in the paper named as Multi-Parametric Sensing by Multi-Channel Molecular Fluorescent Probes Based on Excited State Intramolecular Proton Transfer and Charge Transfer Processes.

## Data Availability

Data will be made available on request.
